# Evaluation of the Anti-inflammatory, Antimicrobial, Antioxidant, and Cytotoxic Effects of Chitosan Thiocolchicoside-Lauric Acid Nanogel

**DOI:** 10.7759/cureus.46003

**Published:** 2023-09-26

**Authors:** Ameena M, Meignana Arumugham I, Karthikeyan Ramalingam, Rajeshkumar S

**Affiliations:** 1 Oral Pathology and Microbiology, Saveetha Dental College and Hospitals, Saveetha Institute of Medical and Technical Sciences, Saveetha University, Chennai, IND; 2 Public Health Dentistry, Saveetha Dental College and Hospitals, Saveetha Institute of Medical and Technical Sciences, Saveetha University, Chennai, IND; 3 Pharmacology, Saveetha Dental College and Hospitals, Saveetha Institute of Medical and Technical Sciences, Saveetha University, Chennai, IND

**Keywords:** lethality assay, cytotoxicity, antimicrobial, lauric acid, thiocolchicoside, chitosan, ctla, antioxidant, anti-inflammatory, nanogel

## Abstract

Aim: The present study explored the anti-inflammatory, antimicrobial, antioxidant, and cytotoxic effects of a combination of chitosan thiocolchicoside and lauric acid (CTLA) nanogel.

Materials and methods: A nanogel formulation of thiocolchicoside and lauric acid was developed and tested for potential applications. The antimicrobial activity was assessed using the well diffusion method, while the antioxidant activity was evaluated using the 2,2-diphenyl-1-picryl hydrazyl (DPPH) free radical scavenging assay and hydrogen peroxide (H_2_O_2_) antioxidant assay methods. The anti-inflammatory activity was determined through the egg albumin denaturation method, the bovine serum albumin denaturation method, and the membrane stabilization assay. A brine shrimp lethality assay was used to study the cytotoxic effect of the nanogel.

Results: We identified significant positive outcomes for the CTLA nanogel. The results showed a percentage of inhibition of 81% at 50μg/mL, which showed the nanogel’s significant anti-inflammatory activity by inhibiting bovine serum albumin denaturation. The anti-inflammatory properties of the nanogel were comparable to the standard diclofenac sodium at all tested concentrations. The egg albumin denaturation assay results revealed a percentage inhibition of 76% at 50 μg/mL. In the membrane stabilization assay, a percentage inhibition of 86% was obtained at a concentration of 50 μg/mL against 89% for the standard drug. The nanogel exhibited a zone of inhibition of 20 mm against *Streptococcus mutans* and 22 mm with a dilution of 100 µg/mL of CTLA nanogel against *Staphylococcus aureus*. The antioxidant activity was studied by using the DPPH method, 50 μg/ml has an 89% inhibition, which was similar to the standard. The inhibitory activity of CTLA nanogel at 50 μg/ml was 81.6% in the hydroxyl free radical scavenging assay, which was comparable to the standard drug. At 5 μg/mL concentration of CTLA nanogel, approximately 90% of the nauplii remained alive after 48 hours.

Conclusion: The CTLA nanogel showed excellent anti-inflammatory and antioxidant properties suggesting its potential for managing inflammatory conditions and oxidative stress-related disorders.

## Introduction

Nanogels are hybrid materials that blend the properties of nanomaterials with hydrogels [1]. Hydrogels, known for their high water content, offer tunability regarding their physical and chemical structures, and excellent mechanical and nontoxic properties [2]. In nanomedicine, nanogel-based formulations have shown great potential in various applications, including imaging, anticancer therapy, and drug delivery [3].

Chitosan, a deacetylated chitin is a biopolymer that is a significant component of the cell wall of fungi, the exoskeleton of insects, and the shells of crustaceans. It is a linear copolymer containing β-(1 to 4)-2-amino-d-glucose units and β-(1 to 4)-2-acetamido-d-glucose units, known to have excellent properties like biodegradability and biocompatibility with the least immune response even after implantation or application due to its nontoxic nature [4].

Thiocolchicoside is a semi-synthetic derivative of colchicoside, which is obtained from plants like *Gloriosa superba* and *Colchicum autumnale* [5]. It is an anti-inflammatory drug used as a muscle relaxant in the treatment of musculoskeletal disorders [6]. The muscle relaxant activity of thiocolchicoside is attributed to its inhibition of the glycine receptor in the brain stem and spinal cord [7]. In previous studies, thiocolchicoside has been formulated in various semi-solid forms such as ointments, gels, and creams, with ointments showing higher drug release compared to other formulations [8].

Lauric acid, on the other hand, is a saturated fatty acid commonly used in nutritional and cosmetic applications. It is a 12-carbon chain fatty acid found in certain plants, particularly coconut oil and palm kernel oil [9]. Lauric acid has been recognized for its broad spectrum of antimicrobial activity against bacteria and viruses. Although the exact mechanism of its action against bacteria is not fully understood, it is believed to disrupt the cell membrane and is thus helpful in protecting against microbial infection and can control human microbiota balance in our body [10,11]. Due to its biological activity and strong antiviral properties, lauric acid is considered one of the most active components in coconut oil [12].

Inflammaging is a term used to denote a systemically developing chronic inflammation that is of low-grade type in the absence of infection in elderly people. As most age-related disorders are linked with inflammation, inflammaging is a significant risk factor for morbidity and mortality in old age [13]. Developing new anti-inflammatory drugs is always of interest to limit the chronic inflammatory process in the human body which includes arthritis, colitis, dermatitis, neurodegenerative disorders, and malignancy. Since the anti-inflammatory drugs to relieve inflammation are always non-steroidal anti-inflammatory drugs (NSAID) or corticosteroids, which develop a lot of adverse drug reactions like gastric irritation, and liver, and renal disorders on long-term usage [14].

Mitochondrial metabolism plays a powerful role in the induction of carcinogenesis by increasing the levels of reactive oxygen species (ROS) production from oncogene transformation to cancer progression. An increase in ROS production results in structural damage to cellular components which leads to cancer, inflammation, and different disorders. There is a recent trend to study natural compounds with significant antioxidant activity that could affect the redox reactions taking place in a cell and prevent and control free radical-mediated reactions [4].

The objective of the present study was to assess the antimicrobial activity, antioxidant activity, anti-inflammatory activity, and cytotoxic effects of a chitosan nanogel formulation containing thiocolchicoside and lauric acid (CTLA nanogel). By combining these two active components, the researchers aimed to explore the potential synergistic effects and broaden the biomedical applications of the nanogel.

## Materials and methods

Preparation of chitosan thiocolchicoside and lauric acid (CTLA) nanogel was reported by Ameena et al. [15]. This method allowed for the integration of the lauric acid and thiocolchicoside into a nanogel formulation. The chitosan served as a medium to facilitate the incorporation of the active components and stabilize the nanogel structure.

Anti-inflammatory activity

Bovine Serum Albumin (BSA) Denaturation Assay

The anti-inflammatory activity of the CTLA nanogel was evaluated as described by Das et al. [16]. To assess the anti-inflammatory activity, 0.05 mL of the CTLA nanogel was taken, and various concentrations ranging from 10µg/mL, 20µg/mL, 30µg/mL, 40µg/mL, and 50µg/mL were added to 0.45 mL of a 1% aqueous solution of bovine serum albumin. The pH of the solution was corrected to 6.3 using a small amount of 1N hydrochloric acid. These samples were then incubated at room temperature for 20 minutes, followed by heating at 55°C for 30 minutes in a water bath. After the heating process, the samples were allowed to cool down, and the absorbance was measured using a spectrophotometer at 660 nm. Diclofenac sodium was the standard drug for comparison. Dimethyl sulfoxide (DMSO) was used as a control in this experiment.

The percentage of protein denaturation was determined using the following equation: % Inhibition = (Absorbance of control - Absorbance of sample/Absorbance of control) x 100.

Egg Albumin Denaturation Assay

The anti-inflammatory activity of the CTLA nanogel was determined. The samples used for this assay include 0.2 mL of egg albumin (fresh), 2.8 mL of phosphate-buffered saline (PBS) at pH 6.4, and 0.6 mL of the nanogel at various concentrations dissolved in 0.2% DMSO. The concentrations of the nanogel in the total reaction solution ranged from 10-50 µg/mL. The samples were incubated for 10 minutes at 37°C and then heated at 70°C in a water bath for an additional 20 minutes to induce denaturation of the egg albumin. After cooling the mixture, the absorbance was measured at 660 nm. Negative controls consisting of 0.2 mL of fresh egg albumin, 0.6 mL of 0.2% DMSO, and 2.8 mL of PBS were included in the experiment. Diclofenac sodium was used as a positive control for the study.

The percentage of protein denaturation inhibition, which indicates the anti-inflammatory activity of the compound, was calculated by the following equation: % Inhibition = (As/Ac - 1) × 100 (As = absorbance of sample, Ac = absorbance of control).

Membrane Stabilization Assay

The in vitro membrane stabilization assay is a commonly employed technique for evaluating the membrane stabilizing properties of natural and synthetic drugs. This assay measures the ability of a drug to stabilize the cell membrane by preventing its disruption and subsequent release of intracellular contents. The materials include human red blood cells (RBCs), Tris-hydrochloride (Tris-HCl) buffer (50 mM at pH 7.4), and PBS. Different concentrations of CTLA nanogel (10-50 µg/mL) were prepared. Saline solution and distilled water were used as controls in this study.

Fresh human blood was collected in a sterile tube containing anticoagulants. The blood was centrifuged for 10 minutes at 1000 g at room temperature to separate the RBCs from other blood components. The supernatant was slowly removed and the RBCs left behind were washed three times using PBS. Then RBCs were resuspended in Tris-HCl buffer to obtain a 10% (v/v) RBC suspension.

1mL of the RBC suspension was pipetted into each centrifuge tube and different concentrations of CTLA nanogel were added to each tube, gently mixed, and incubated for 30 minutes at 37°C. The centrifuge tubes were then centrifuged at 1000 g for 10 minutes at room temperature to pellet the RBCs. The absorbance of the supernatant obtained was measured at 540 nm using an ultraviolet spectrophotometer.

The percentage inhibition of hemolysis was calculated using the following formula: % Inhibition = {(OD control - OD sample)/OD control} x 100. OD control in the formula is the absorbance of the RBC suspension without the test compound and OD sample is the absorbance of the RBC suspension with the test compound.

Anti-microbial activity

The antibacterial activity of the CTLA nanogel was investigated against bacterial strains including *Streptococcus mutans*, *Staphylococcus aureus*, *Pseudomonas aeruginosa*, *Lactobacillus *and *Candida albicans*. For this experiment, a 24-hour freshly prepared culture of the bacteria was used. To determine the zone of inhibition, Muller-Hinton agar (MHA) was prepared and sterilized by autoclaving at 121°C for 30 minutes. The sterilized MHA was poured into sterile Petri plates and was allowed to solidify. The wells were then created using a Well cutter and the fresh bacterial culture of *Streptococcus mutans*, *Staphylococcus aureus*, *Pseudomonas aeruginosa*, *Lactobacillus *and *Candida albicans* was evenly spread on the Petri plates. Different concentrations of the CTLA nanogel (25 µg, 50 µg, 100 µg) were loaded into separate wells on the agar plate in triplicates. Additionally, an antibiotic Amoxyrite was used as a standard for bacteria, and for candida, Fluconazole was used as a standard and placed in the fourth well for comparison. The plates were incubated for 24 hours and 48 hours for fungal cultures at 37°C. The antimicrobial activity of the compound was assessed by measuring the diameter of the zone of inhibition around the wells. The zone of inhibition was measured using a ruler and recorded in millimeters (mm). This measurement indicated the antibacterial effectiveness of the nanogel.

Anti-oxidant activity

2,2-Diphenyl-1-Picryl Hydrazyl (DPPH) Free Radical Scavenging Assay

The antioxidant activity of CTLA nanogel was analyzed using the DPPH assay. Various concentrations (10μg/mL, 20 μg/mL, 30 μg/mL, 40 μg/mL, and 50 μg/mL) of the nanogel were mixed with 1 mL of DPPH (0.1 mM) in methanol and 450 μg/mL of 50 mM Tris-HCl buffer at pH 7.4. The mixture was then incubated in a dark room for 30 minutes. The reduction in the quantity of the DPPH free radical was assessed by measuring the absorbance at 517 nm. This measurement indicated the antioxidant capacity of the nanogel. Ascorbic acid was used as standard in this assay. By evaluating the absorbance at 517 nm, this assay determined the antioxidant activity of the CTLA nanogel.

The percentage of the inhibition was determined from the following equation: % inhibition of sample = (Absorbance of control - Absorbance of sample/Absorbance of control) x 100.

Hydroxyl Free Radical Scavenging Assay

The hydroxyl free radical scavenging assay was conducted. Freshly prepared solutions were used for the experiment. In a reaction mixture of 1.0 mL, the following components were added: 100 µL of a 28 mM solution of 2-deoxy-2-ribose dissolved in phosphate buffer at a pH 7.4, 500 µL of a solution containing different concentrations of the CTLA nanogel (ranging from 10 to 50 µg), 200 µL of a 200 µM ferric chloride (FeCl_3_) and 1.04 mM ethylenediaminetetraacetic acid (EDTA) mixture in a 1:1 volume ratio, 100 µL of H_2_O_2_ (1.0 mM), and 100 µL of ascorbic acid (1.0 mM). The reaction mixture was incubated for 1 hour at 37°C. The extent of deoxyribose degradation after the incubation period, was determined by thiobarbituric acid (TBA) reaction. The mixture was further incubated for 1 hour at 37°C, and the optical density at 532 nm was measured against a blank solution. For comparison, vitamin E served as the positive control, and ascorbic acid was used as the standard. By measuring the optical density at 532 nm, the hydroxyl radical scavenging activity of the CTLA nanogel was assessed.

Cytotoxic effect

Brine Shrimp Lethality Assay

To prepare the solution, 2 grams of iodine-free salt was weighed and dissolved in 200 mL of distilled water. Six enzyme-linked immunosorbent Assay (ELISA) well plates were used for the experiment, and each well was filled with 10-12 mL of prepared saline water. Subsequently, 10 nauplii were added slowly to each well, with different concentrations of the CTLA nanogel (5 µg/mL, 10 µg/mL, 20 µg/mL, 40 µg/mL, and 80 µg/mL) added to the respective wells. The sixth well served as a control and did not receive the nanogel. The plates were then incubated at room temperature for 24 hours, allowing the desired effects of the nanogel on the nauplii to take place.

After 24 hours, the ELISA plates were carefully observed and counted for the number of live nauplii present and calculated by using the following formula: Number of dead nauplii/Number of dead nauplii + Number of live nauplii × 100.

## Results

The anti-inflammatory activity of CTLA nanogel was evaluated using the bovine serum albumin denaturation assay. The nanogel was tested at different concentrations, and its inhibitory effects were compared to standard values. The results showed a percentage of inhibition of 47% at a concentration of 10 μg/mL, 53% at 20 μg/mL, 69% at 30 μg/mL, 72% at 40 μg/mL, and 81% at 50 μg/mL. These values indicate that the nanogel exhibits significant anti-inflammatory activity by inhibiting bovine serum albumin denaturation. Moreover, the anti-inflammatory properties of the nanogel were comparable to the standard diclofenac sodium at all tested concentrations (Table 1).

**Table 1:** Anti-inflammatory activity with BSA assay Table showing the anti-inflammatory activity of chitosan thiocolchicoside lauric acid (CTLA) nanogel with bovine serum albumin (BSA) denaturation assay

	10 μg/mL	20 μg/mL	30 μg/mL	40 μg/mL	50 μg/mL
CTLA nanogel	44	58	70	75	80
Standard	47	60	72	78	84

The anti-inflammatory activity of CTLA nanogel was assessed using the egg albumin denaturation assay. The nanogel was tested at various concentrations and compared to standard values. The results revealed a percentage of inhibition of 53% at a concentration of 10 μg/mL, 58% at 20 μg/mL, 61% at 30 μg/mL, 69% at 40 μg/mL, and 76% at 50 μg/mL. These findings demonstrate that the nanogel exhibits significant anti-inflammatory activity in the egg albumin denaturation assay. Furthermore, the anti-inflammatory properties of the nanogel were found to be comparable to the standard diclofenac sodium at all tested concentrations like 10 μg/mL, 20 μg/mL, 30 μg/mL, 40 μg/mL, and 50 μg/mL. Therefore, the CTLA nanogel shows promising anti-inflammatory activity in the context of the egg albumin denaturation assay (Table 2).

**Table 2:** Anti-inflammatory activity with egg albumin assay Table showing the anti-inflammatory activity of chitosan thiocolchicoside lauric acid (CTLA) nanogel with egg albumin denaturation assay

	10 μg/mL	20 μg/mL	30 μg/mL	40 μg/mL	50 μg/mL
CTLA nanogel	53	62	66	70	80
Standard	55	64	69	72	81

The anti-inflammatory activity of CTLA nanogel was assessed using the human red blood cell membrane stabilization assay. The nanogel was tested at different concentrations and compared to standard values. The results revealed a percentage of inhibition of 56% at a concentration of 10 μg/mL, 67% at 20 μg/mL, 75% at 30 μg/mL, 80% at 40 μg/mL, and 86% at 50 μg/mL against 58%, 70%, 77%, 82%, and 89% for the standard, diclofenac sodium at same concentrations. These findings show a significant anti-inflammatory activity of CTLA nanogel in the membrane stabilization assay (Table 3).

**Table 3:** Red blood cell membrane stabilization assay Table showing the human red blood cell membrane stabilization assay of chitosan thiocolchicoside lauric acid (CTLA) nanogel

	10 μg/mL	20 μg/mL	30 μg/mL	40 μg/mL	50 μg/mL
CTLA nanogel	56	67	75	80	86
Standard	58	70	77	82	89

**Figure 1:**
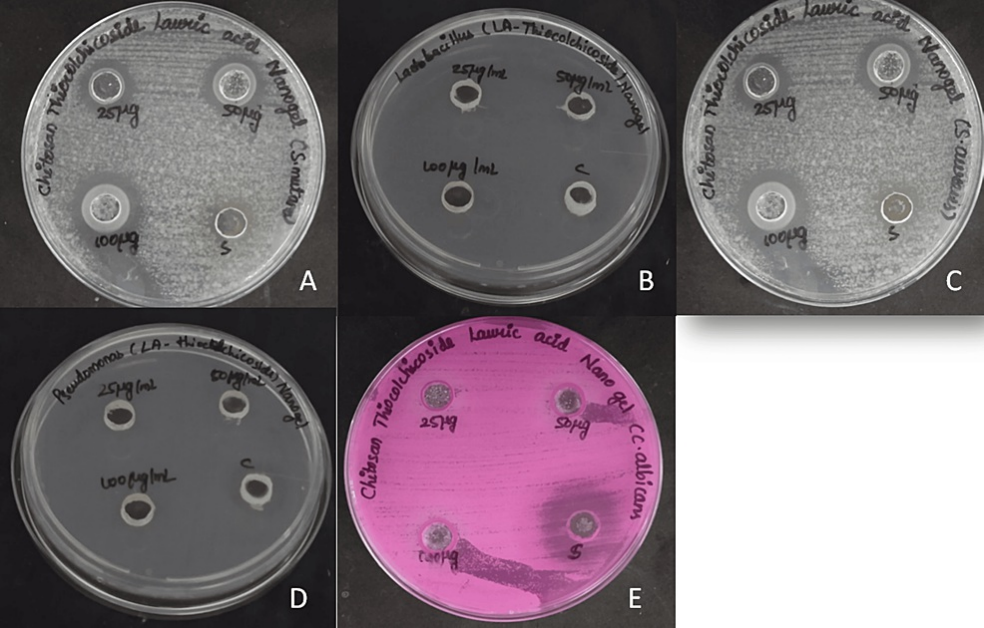
Antimicrobial activity of CTLA nanogel Images showing the anti-microbial activity of chitosan thiocolchicoside lauric acid (CTLA) nanogel against *Streptococcus mutans* (A), *Lactobacillus* species (B), *Staphylococcus aureus* (C), *Pseudomonas aeruginosa* (D), and *Candida albicans* (E)

The antimicrobial activity of CTLA nanogel was evaluated using the agar well diffusion method. The nanogel exhibited a zone of inhibition of 20 mm with a dilution of 100 µg/mL of CTLA nanogel against *Streptococcus mutans* while 13 mm was the zone of inhibition for the standard. The nanogel developed a zone of inhibition of 22 mm with a dilution of 100 µg/mL of CTLA nanogel against *Staphylococcus aureus* while 11 mm was the zone of inhibition for the standard. The zone of inhibition of CTLA nanogel at 100 µg/mL against *Candida albicans* was 16 mm and fluconazole was 20 mm. The nanogel exhibited a zone of inhibition of 9 mm against both *Lactobacillus* species and *Pseudomonas aeruginosa* at three different concentrations, indicating similar levels of inhibition for both bacterial strains. In comparison, the fourth well, which contained the commercial antibiotic Amoxyrite, showed a higher zone of inhibition of 14 mm against *Lactobacillus* species and a lower area of inhibition of 9 mm against *Pseudomonas aeruginosa* at the same diluted concentration (Figure 1).

**Figure 2:**
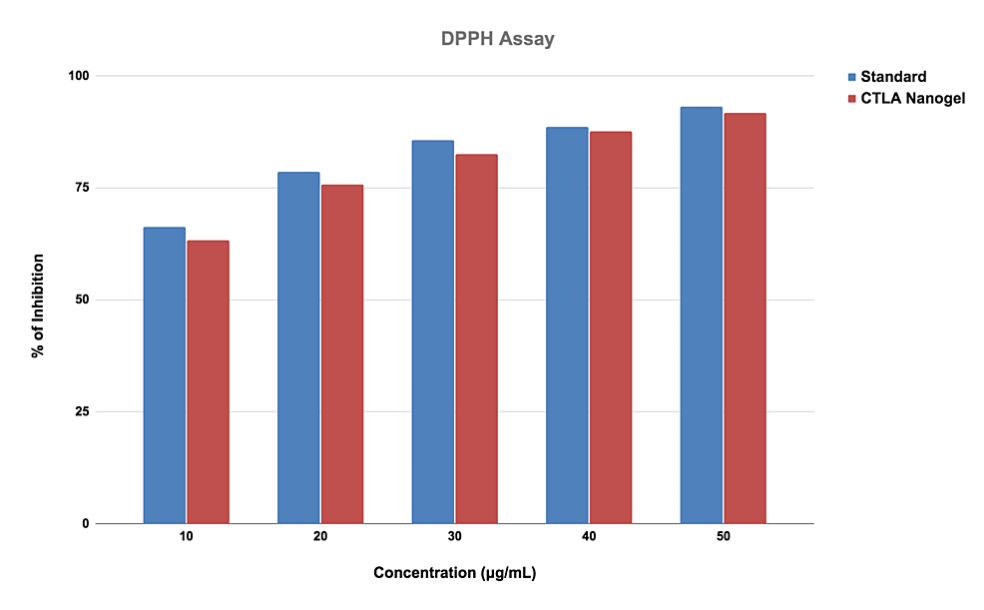
Graph of DPPH assay Graphical representation of the activity of chitosan thiocolchicoside lauric acid (CTLA) nanogel estimated by 2,2-diphenyl-1-picryl hydrazyl (DPPH) free radical scavenging assay

The antioxidant activity was done by using the DPPH method. DPPH assay was compared from a lower concentration to a higher concentration of the CTLA nanogel. In the CTLA nanogel, a different concentration was added. In the different concentrations of nanogel, 10μg/ml had a 68% inhibition, 20 μg/ml had a 76% inhibition, 30 μg/ml had a 79% inhibition, 40 μg/ml had an 85% of inhibition and 50 μg/ml had an 89% of inhibition. The antioxidant activity of CTLA nanogel was slightly similar when compared to the standard (Figure 2).

**Figure 3:**
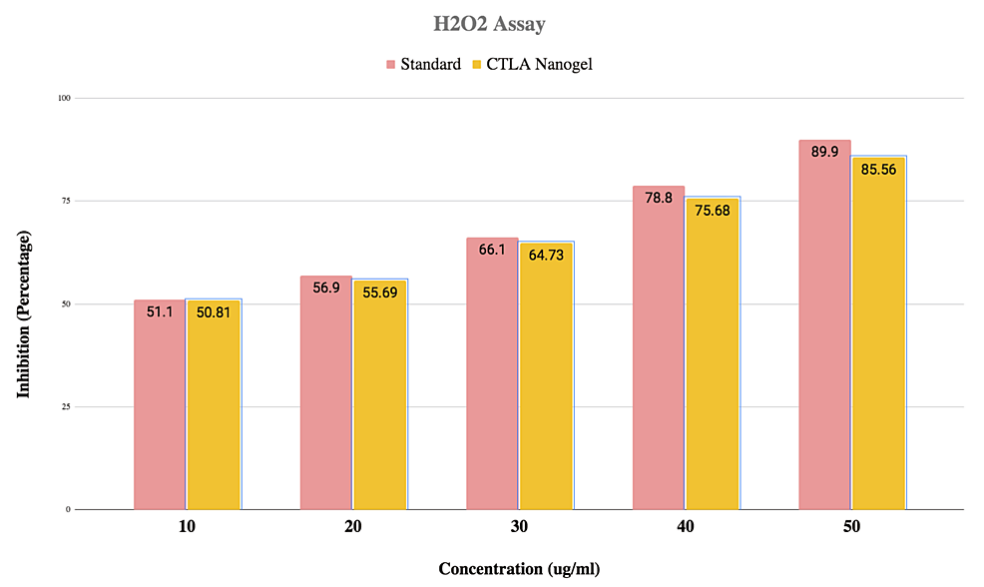
Graph of H2O2 assay Graphical representation of the activity of chitosan thiocolchicoside lauric acid (CTLA) nanogel estimated with hydrogen peroxide (H2O2) assay

The antioxidant activity of CTLA nanogel was evaluated using the H_2_O_2_ method. Different nanogel concentrations were tested, ranging from lower to higher concentrations. The results showed that the nanogel exhibited antioxidant activity, with increasing levels of inhibition as the concentration of the nanogel increased. At 10 μg/ml, the nanogel demonstrated 50.6% inhibition, while at 20 μg/ml, it showed 54.7% inhibition. The inhibitory activity further increased at 30 μg/ml with 65.12% inhibition, at 40 μg/ml with 74.3% inhibition, and at 50 μg/ml with 81.6% inhibition. The CTLA nanogel displayed similar antioxidant activity to the standard (Figure 3).

**Figure 4:**
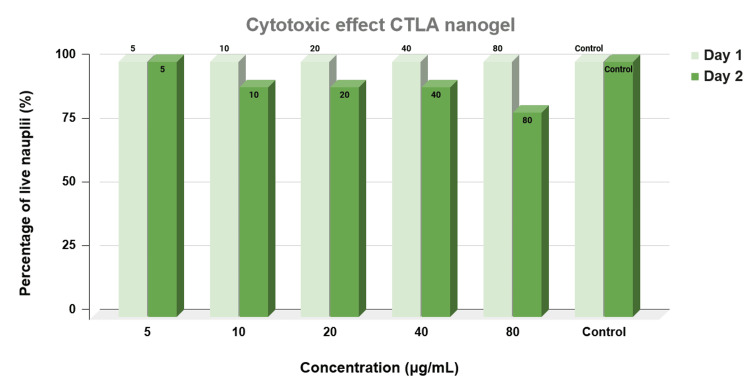
Graph showing the cytotoxic effect of CTLA nanogel Graphical representation of the cytotoxic effect of chitosan thiocolchicoside lauric acid (CTLA) nanogel estimated by brine shrimp lethality assay

The cytotoxic effects of CTLA nanogel were evaluated using the brine shrimp lethality assay. This assay is commonly employed to assess the cytotoxicity of substances by measuring their impact on the survival of the brine shrimp nauplii. In the present study, a control group without any drug was maintained to establish a baseline for calculating the percentage of live nauplii. The results of the cytotoxicity assessment indicated that different concentrations of the nanogel exhibited varying effects on nauplii survival. At a concentration of 5 μg/mL of thiocolchicoside-lauric acid nanogel, approximately 90% of the nauplii remained alive. Similarly, at concentrations of 20 μg/mL and 40 μg/mL, the nanogel resulted in the preservation of approximately 70% of live nauplii. However, at a higher concentration of 80 μg/mL, only 60% of the nauplii survived (Figure 4).

## Discussion

Anti-inflammatory properties of various plant extracts are available in the literature [17]. Overall, our study highlights the ability of the CTLA nanogel and the herbal formulation extract to effectively inhibit inflammation by preventing protein denaturation.

The lysosomal membrane stabilization in activated neutrophils prevents the leakage of lysosomal contents like protease and other bactericidal enzymes, thereby playing a pivotal role in anti-inflammatory response in the human body. Red blood cells and lysosomal membranes of neutrophils have a similar structure, so stabilization of one membrane may limit the destruction of the other. This principle is the basis for the human red blood cell membrane stabilization assay which uses hypotonicity-induced lysis to evaluate the anti-inflammatory activity of drugs [14]. In the present study, it was found that the CTLA nanogel had excellent anti-inflammatory action using the human red blood cell membrane stabilization assay, and the process of membrane stabilization could be directly related to its anti-inflammatory properties.

Kyene et al. using the agar well diffusion method studied the antimicrobial activity of the zinc oxide nano particles against *Staphylococcus aureus*, *Escherichia coli*, *Salmonella typhi*, and *Candida albicans* [18]. Shanmugam et al. reported that silver nanoparticles with the addition of curcumin-assisted chitosan nanocomposite had remarkable antibacterial activity against gram-positive bacteria when compared to gram-negative bacteria in their study [19].

Furthermore, the individual component of the nanogel, lauric acid, demonstrated significant inhibitory effects against various clinical isolates. In previous research works, the inhibitory effect varied based on the concentration of the acid, with the highest zone of inhibition observed against *Staphylococcus aureus* (10 mm), *Streptococcus *species (10 mm), and *Lactobacillus* species (10 mm). On the other hand, the lowest inhibitory effect was found against *Escherichia coli* (4 mm) at the same concentration of dilution [12]. Previous studies have also reported the antimicrobial effects of lauric acid, specifically against Gram-positive streptococci but not as effective against Gram-negative bacilli such as *Escherichia coli*, *Klebsiella oxytoca*, *Klebsiella* *pneumoniae*, and *Serratia marcescens* [11]. Nagase et al. compared the bactericidal activity of ovirgin coconut oil and lauric acid using an antibacterial disk diffusion test and reported that the bacteria-inhibiting zone was 0.17 μmol /40 μL or 0.085 μmol / 40 μL of Lauric acid on plates inoculated with *Streptococcus pyogenes*, *Streptococcus agalactiae*, *Streptococcus mutans*, and *Streptococcus sanguinis* and was greater than that of the paper disk containing 0.17 μ mol40 μL of virgin coconut oil [20]. They found that lauric acid has significant antimicrobial activity against *Staphylococcus aureus* and *Streptococcus salivarius* and organic virgin coconut oil had weaker antimicrobial activities against several *Streptococcus* species than lauric acid.

Bhardwaj et al. studied the antibacterial activity of coconut oil by agar well diffusion method using ciprofloxacin as a standard antibiotic [21]. They found that *Streptococcus* species were susceptible to coconut oil while *Escherichia coli *were not. They confirmed that the presence of lauric acid is responsible for the bactericidal action of coconut oil. These findings highlight the antimicrobial potential of lauric acid, particularly against certain bacterial strains, as demonstrated by various studies.

Chitosan nanoparticles exhibited redox-regulatory activity due to inhibition of free radical production, decreasing serum free fatty acids, and malondialdehyde, and increasing intracellular antioxidant enzymes in vitro as well as in vivo studies [4]. The antioxidant activity of CTLA nanogel was evaluated using the DPPH method. Different nanogel concentrations were tested, ranging from lower to higher concentrations. The results indicated that the nanogel exhibited antioxidant activity, with varying degrees of inhibition at each concentration. Comparatively, the CTLA nanogel showed a slight similarity to the standard antioxidant.

Virgin coconut oil reduced the DPPH free radical concentration by 50% and EC50 was found to be 5.07 ± 0.19 mg/L in a study by Ahmad et al. [22]. The hydrogen-donating capacity of virgin coconut oil makes it a good antioxidant, but the level of free-radical scavenging activity depends on the processing condition of virgin coconut oil [22]. The free radical scavenging activity (%) of green tea-loaded with chitosan nanoparticles, green tea, and ascorbic acid was reported by Piran et al. showed the high antioxidant activity of green tea and green tea-loaded chitosan nanoparticles [23]. The range of green tea and green tea-loaded chitosan nanoparticles scavenging was found to be 32.07-91.0  μg/ml and 46.75-96.1 μg/ml respectively [23]. Wen et al. observed the effects of chitosan nanoparticles in H2O2-induced oxidative damage in murine macrophage cells and found that viability loss in cells induced by H2O2 was significantly replaced by chitosan nanoparticles [24]. It suppressed the production of malondialdehyde, restored superoxide dismutase and glutathione peroxidase, and increased total antioxidant capacity.

Marina et al. reported that virgin coconut oil, due to its antioxidant properties, reduced the initial concentration of DPPH radicals by 50%, with an EC50 value of approximately 5.07 ± 0.19 mg/L [25]. Virgin coconut oil can donate hydrogen ions and thus can act as an antioxidant. The processing conditions of virgin coconut oil may influence the free radical scavenging activity of its phenolic compounds [25].

Thyme essential oil encapsulated in chitosan nanoparticles proved to have greater antioxidant activity than free thyme essential oil [26]. Similarly, green tea-loaded chitosan nanoparticles and green tea itself demonstrated significant scavenging activity, indicating high antioxidant capacity. The scavenging activity ranged from 32.07% to 91.034% for green tea and from 46.75% to 96.12% for green tea-loaded chitosan nanoparticles at various concentrations [27]. Overall, the CTLA nanogel, exhibited promising antioxidant properties, as demonstrated by their ability to scavenge free radicals and inhibit oxidative processes.

Overall, the cytotoxic activity results revealed a relatively low toxicity rate of the CTLA nanogel, which aligns with the findings of the current study. This indicates that the nanogel formulation exhibited minimal cytotoxic effects on brine shrimp nauplii, suggesting its potential safety for future applications.

Limitations

We performed various in-vitro analyses in the present study to assess the activity of CTLA nanogel. Testing with Fourier-transform infrared spectroscopy (FTIR) or nuclear magnetic resonance (NMR) will help to decipher the active ingredients of our nanogel formulation. Further in vivo studies including animal studies and clinical trials will help in a better understanding of its effects.

## Conclusions

In conclusion, combining thiocolchicoside and lauric acid as chitosan nanogel showcases a versatile therapeutic agent with multifaceted properties. Its antimicrobial activity, eco-friendly nature, and biocompatibility highlight its promising role in combating infections. Moreover, its anti-inflammatory and antioxidant properties suggest its potential for managing inflammatory conditions and oxidative stress-related disorders. These characteristics position thiocolchicoside-lauric acid as a promising candidate for future biomedical applications, paving the way for further exploration and development in the field of medicine.

## References

[1] Hamidi M, Azadi A, Rafiei P (2008). Hydrogel nanoparticles in drug delivery. Adv Drug Deliv Rev.

[2] Ahmed EM (2015). Hydrogel: preparation, characterization, and applications: a review. J Adv Res.

[3] Molina M, Asadian-Birjand M, Balach J, Bergueiro J, Miceli E, Calderón M (2015). Stimuli-responsive nanogel composites and their application in nanomedicine. Chem Soc Rev.

[4] Ivanova DG, Yaneva ZL (2020). Antioxidant properties and redox-modulating activity of chitosan and its derivatives: biomaterials with application in cancer therapy. Biores Open Access.

[5] Mahendran D, Kavi Kishor PB, Sreeramanan S, Venkatachalam P. (2018). Enhanced biosynthesis of colchicine and thiocolchicoside contents in cell suspension cultures of Gloriosa superba L. exposed to ethylene inhibitor and elicitors. Ind Crops Prod.

[6] Cimino M, Marini P, Cattabeni F (1996). Interaction of thiocolchicoside with [3H]strychnine binding sites in rat spinal cord and brainstem. Eur J Pharmacol.

[7] Shrivastav J, Shah K, Mahadik M (2011). Application of HPTLC in the simultaneous estimation of thiocolchicoside and diclofenac in bulk drug and pharmaceutical dosage form. Bull Pharm Res.

[8] Jiang L, Liang X, Liu G (2018). The mechanism of lauric acid-modified protein nanocapsules escape from intercellular trafficking vesicles and its implication for drug delivery. Drug Deliv.

[9] Rozanna D, Chuah TG, Salmiah A (2005). Fatty acids as phase change materials (PCMs) for thermal energy storage: a review. Int J Green Energy.

[10] Ogbolu DO, Oni AA, Daini OA (2007). In vitro antimicrobial properties of coconut oil on Candida species in Ibadan, Nigeria. J Med Food.

[11] Matsue M, Mori Y, Nagase S (2019). Measuring the Antimicrobial Activity of Lauric Acid against Various Bacteria in Human Gut Microbiota Using a New Method. Cell Transplant.

[12] Abbas A, Assikong EB, Akeh M, Upla P (2017). Antimicrobial activity of coconut oil and its derivative (lauric acid) on some selected clinical isolates. Int J Med Sci Clin Invent.

[13] Shayganni E, Bahmani M, Asgary S, Rafieian-Kopaei M (2016). Inflammaging and cardiovascular disease: management by medicinal plants. Phytomedicine.

[14] Qamar M, Akhtar S, Ismail T (2021). Syzygium cumini (L.), skeels fruit extracts: in vitro and in vivo anti-inflammatory properties. J Ethnopharmacol.

[15] Ameena M, Meignana Arumugham I, Ramalingam K, Rajeshkumar S, Perumal E (2023). Cytocompatibility and Wound Healing Activity of Chitosan Thiocolchicoside Lauric Acid Nanogel in Human Gingival Fibroblast Cells. Cureus.

[16] Das P, Ghosal K, Jana NK, Mukherjee A, Basak P (2019). Green synthesis and characterization of silver nanoparticles using belladonna mother tincture and its efficacy as a potential antibacterial and anti-inflammatory agent. Mater Chem Phys.

[17] Pranati T, Rajasekar A, Rajeshkumar S (2020). Anti-inflammatory and cytotoxic effect of clove and cinnamon herbal formulation. Plant Cell Biotechnol Mol Biol.

[18] Kyene MO, Droepenu EK, Ayertey F (2023). Synthesis and characterization of ZnO nanomaterial from Cassia sieberiana and determination of its anti-inflammatory, antioxidant and antimicrobial activities. Sci African.

[19] Shanmugam R, Subramaniam R, Kathirason SG (2021). Curcumin-chitosan nanocomposite formulation containing Pongamia pinnata-mediated silver nanoparticles, wound pathogen control, and anti-inflammatory potential. Biomed Res Int.

[20] Nagase S, Matsue M, Sugitani K (2017). Comparison of the antimicrobial spectrum and mechanisms of organic virgin coconut oil and lauric acid against bacteria (Article in Japanese). J Wellness Heal Care.

[21] Bhardwaj V (2023). Antimicrobial potential of Cocos nucifera (coconut) oil on bacterial isolates. Adv Exp Med Biol.

[22] Ahmad Z, Hasham R, Nor NF, Sarmidi MR (2015). Physico-chemical and antioxidant analysis of virgin coconut oil using West African tall variety. J Adv Res in Materials Science.

[23] Piran F, Khoshkhoo Z, Hosseini SE, Azizi MH (2020). Controlling the antioxidant activity of green tea extract through encapsulation in chitosan-citrate nanogel. J Food Qual.

[24] Wen ZS, Liu LJ, Qu YL, Ouyang XK, Yang LY, Xu ZR (2013). Chitosan nanoparticles attenuate hydrogen peroxide-induced stress injury in mouse macrophage RAW264.7 cells. Mar Drugs.

[25] Marina AM, Man YB, Nazimah SA, Amin I (2009). Antioxidant capacity and phenolic acids of virgin coconut oil. Int J Food Sci Nutr.

[26] Ghaderi GM, Barzegar M, Sahari MA, Azizi MH. (2016). Enhancement of thermal stability and antioxidant activity of thyme essential oil by encapsulation in chitosan nanoparticles. J Agric Sci Technol.

[27] Kedare SB, Singh RP (2011). Genesis and development of DPPH method of antioxidant assay. J Food Sci Technol.

